# Systematic Review of Interventions to Optimize Emergency Department Care of Patients with Cancer

**DOI:** 10.5811/westjem.49006

**Published:** 2026-02-22

**Authors:** Jason G.A. den Duijn, Monica Muharam, Maarten F.M. Engel, Rob J.C.G. Verdonschot, Nick Wlazlo, Gerrie Prins-van Gilst, Monique E.M.M. Bos, Jelmer Alsma

**Affiliations:** * Erasmus MC Cancer Institute, University Medical Center Rotterdam, Department of Medical Oncology, Rotterdam, The Netherlands; † Erasmus MC Cancer Institute, University Medical Center Rotterdam, Medical Library, Rotterdam, The Netherlands; ‡ Erasmus MC, University Medical Center Rotterdam, Department of Emergency Medicine, Rotterdam, The Netherlands; § Erasmus MC Cancer Institute, Erasmus University Medical Center, Department of Hematology, Rotterdam, The Netherlands; || Erasmus MC, University Medical Center Rotterdam, Department of Internal Medicine, Rotterdam, The Netherlands

## Abstract

**Introduction:**

Approximately 12% of patients with cancer annually visit the emergency department (ED) for disease- or treatment-related issues. These patients often face delays in care, including prolonged wait times and extended length of stay (LOS), contributing to ED crowding, delayed treatment, and increased mortality. Numerous studies have investigated interventions to reduce LOS and prevent ED visits for patients with cancer. However, a systematic overview of these interventions is currently lacking. In this review we aimed to present interventions that optimize input, throughput and output in ED care by reducing ED LOS or ED visits for patients with cancer.

**Methods:**

We searched five electronic library databases: Medline ALL via Ovid; Embase.com; Web of Science Core Collection; the Cochrane Central Register of Controlled Trials via Wiley; and Google Scholar. Inclusion criteria for this review were as follows: 1) research on (a subset of) patients with cancer; 2) conducted in or in collaboration with the ED; 3) the introduction of an intervention aimed at optimizing ED input, throughput, and output; and 4) performance of the intervention was measured using outcomes, such as ED LOS, number of ED visits or hospitalizations, use of acute-care services, or time to antibiotics.

**Results:**

The literature search yielded 11,357 articles. After removing duplicates, 7,315 unique articles remained for screening. Of these, 109 were selected for detailed abstract review. Following this second screening, 35 articles underwent full-text analysis, and 16 articles met all inclusion criteria. These studies identified four categories of interventions: scoring systems (n=5); dedicated cancer urgent care facilities (n=5); protocolized care (n=3); and staffing optimization (n=3). Among scoring systems, use of the Edmonton Symptom Assessment Scale reduced ED visits (relative rate (RR) = 0.92) and hospitalizations (RR = 0.86), while the Clinical Index of Stable Febrile Neutropenia score showed higher specificity (98.3%) than the Multinational Association for Supportive Care in Cancer score (54.2%) for identifying low-risk febrile neutropenia.

**Conclusion:**

We identified four categories of intervention that could potentially reduce ED visits and ED LOS, of which scoring systems showed the most potential. Rather than developing new tools, future efforts should prioritize the implementation, validation, and refinement of these existing strategies to optimize treatment of cancer patients in the emergency department.

## INTRODUCTION

Annually, 10–12% of patients with cancer present to the emergency department (ED) with complaints directly related to their disease or treatment.^1^ Of all patients who visit the ED, 5.4% are undergoing cancer treatment.^2^,^3^ In this group, ED length of stay (LOS) is typically prolonged.^4^ This can be attributed to delays in throughput and output, which result from waiting for test results and a more complex decision-making process. A prolonged LOS combined with increased patient volume contributes to crowding, defined as a situation in which the demand for emergency services exceeds the available resources in the ED.^5^ Crowding also contributes to delayed treatment, poorer patient outcomes, and increased mortality rates.^4^,^6^,^7^ Consequently, several strategies have been developed to improve input, throughput, and output for patients requiring acute care.

Given these challenges, numerous studies have investigated interventions designed to reduce ED LOS or to prevent ED visits from patients with cancer. In this systematic review we aimed to provide an overview of interventions that optimize ED input, throughput and output (optimizing intervention) by reducing ED LOS or ED visits in patients with cancer.

## METHODS

This systematic review followed the Preferred Reporting Items for Systematic reviews and Meta-Analyses (PRISMA) checklist and the PRISMA-S extension to the PRISMA Statement for Reporting Literature Searches in Systematic Reviews.^8^,^9^ This study was prospectively registered at PROSPERO (ID: CRD42023434667).

### Search Strategy

An information specialist (ME) and the lead author (JD) designed an Embase.com search optimized for sensitivity and translated to other databases using the method described by Bramer et al.^10^ We searched Medline ALL via Ovid (1946 to Daily Update), Embase.com (1971–present), Web of Science Core Collection (Science Citation Index Expanded (1975–present); Social Sciences Citation Index (1975–present); Arts & Humanities Citation Index (1975–present); Conference Proceedings Citation Index–Science (1990–present); Conference Proceedings Citation Index–Social Science & Humanities (1990–present) and Emerging Sources Citation Index (2015–present), and the Cochrane Central Register of Controlled Trials via Wiley (1992–present). Google Scholar was also searched, and we downloaded the top 200 results using Publish or Perish software (Herzing.com).^11^,^12^ The first search was conducted on 29 June 2023 and last updated on 12 July 2024.

MEDLINE and Embase strategies incorporated Medical Subject Headings and Emtree terms, respectively. Across all databases, papers were searched by title, abstract, and keywords. The search contained terms for 1) emergency department/acute care; 2) patients with cancer; 3) utilization of healthcare/waiting time. We linked terms with Boolean operators (AND, OR) and proximity operators to form phrases. Complete search strategies are available in Appendix 1. Searches excluded conference papers and non-English language papers in every database. We did not search trial registries. However, Cochrane CENTRAL retrieves contents of ClinicalTrials.gov and the World Health Organization’s International Clinical Trials Registry Platform. We screened reference lists of non-included but relevant reviews, included studies, and citing articles for additional records using the methods described by Bramer et al.^13^,^14^ We did not contact authors or experts, nor did we hand-search unindexed journals.

**Population Health Research Capsule**

What do we already know about this issue?

*Cancer patients frequently visit the ED, facing prolonged stays, delays in care, and increased mortality, with various interventions tested but no systematic overview*.

What was the research question?

*What ED interventions reduce cancer patient ED length of stay or visits, and improve care efficiency?*

What was the major finding of the study?

*This review identifies four categories of research that could potentially reduce emergency department visits and length of stay for patients with cancer*.

How does this improve population health?

*Prioritizing existing ED interventions, like scoring systems, can reduce avoidable visits, ED crowding, and improve timeliness and safety of cancer care*.

An information specialist (ME) imported all references into EndNote and deduplicated them using the method as per Bramer et al.^15^

### Inclusion and Exclusion Criteria

We included articles if they met the following criteria: 1) research on (a subset of) patients with cancer; 2) conducted in or in collaboration with the ED; 3) the introduction of an intervention aimed at optimizing ED input, throughput, and output; and 4) performance of the intervention was measured using outcomes such as ED LOS, number of ED visits or hospitalizations, use of acute care services, or time to antibiotics. We excluded abstracts that were published alone as well as full papers that were not publicly available or peer-reviewed, or were published in a language other than English. Also excluded were literature reviews or research that focused on economic, pharmacological, surgical, pediatric, and palliative interventions.

### Screening Process

We managed and screened citations using the artificial intelligence-powered screening application Rayyan (Qatar Computing Research Institute, Ar-Rayyan, Qatar).^16^ Screening involved four steps: 1) titles and abstract were screened for keywords; 2) abstracts were assessed against inclusion criteria; 3) full texts were reviewed for final inclusion or exclusion; and 4) data were extracted from included papers. Two independent researchers (JD/MM) conducted the screening. A third researcher (JA) resolved any disagreements.

### Data Extraction and Synthesis

From each selected paper, we extracted the following elements: author; publication year; country; patient group; population size; study design; the intervention introduced; aim; primary (and secondary) outcome(s); outcome-related results; and level of evidence (I to V, per Elsevier criteria^17^). Given the heterogeneity of study designs, populations, and outcome measures, a quantitative synthesis (meta-analysis) was not feasible. Therefore, we used a narrative synthesis approach, grouping findings by intervention category.

### Quality Assessment

Two reviewers (JD/MM) assessed the included articles using the Quality Assessment with Diverse Studies (QuADS) criteria.^18^ This method evaluates studies with different designs across 13 criteria, each scored from zero to three. It does not include a cut-off score for high or low quality, as it is not intended for that purpose.^18^

## RESULTS

**Figure 1:**
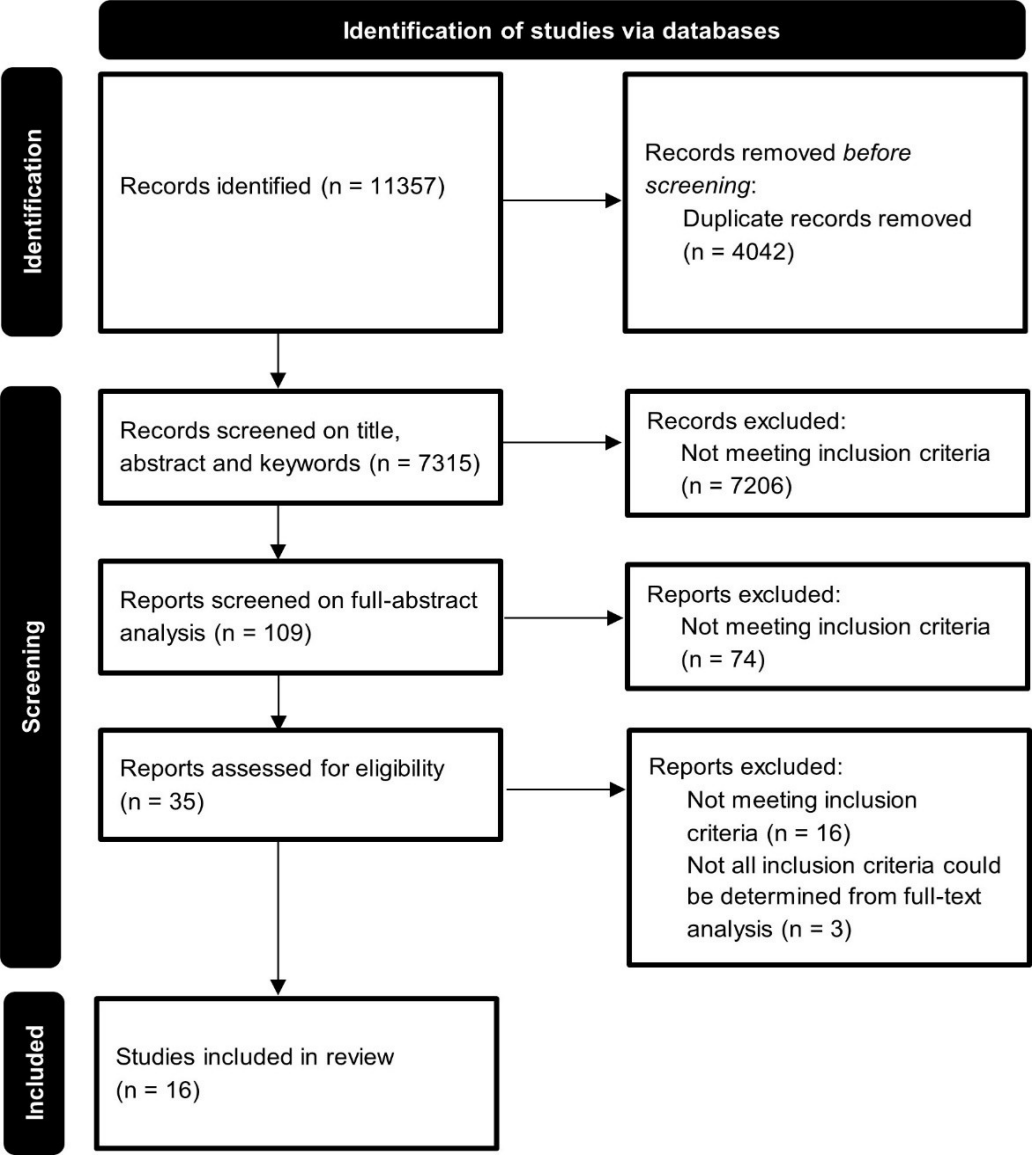
Flow chart of study inclusion in a review of the literature on interventions that optimize input, throughput, and output in emergency department care by reducing length of stay or number of visits for patients with cancer.

The initial search yielded 11,357 papers (1977–2023). After removing duplicates, 7,315 unique papers remained for screening by title, abstract and keyword. From these, 109 papers were selected for a detailed abstract review. After this second screening, 35 papers underwent full-text analysis, of which 16 met all inclusion criteria (Figure 1). We identified four categories after data abstraction: 1) scoring systems (five studies introduced or validated tools to stratify patients with cancer by their risk of requiring emergency care, either before ED presentation or after triage); 2) dedicated cancer urgent care facilities (five studies described the establishment of separate EDs dedicated to patients with cancer); 3) protocolized care (three studies evaluated standardized care through treatment protocols for febrile neutropenia); 4) staffing optimization. (three studies focused on staff-targeted interventions). Table 1 summarizes the study characteristics and outcomes. Table 2 presents the QuADS quality scores, which range from 18–32.

### Scoring Systems

Five studies evaluated scoring systems that stratified patients with cancer by risk of ED visit or hospital admission.^19^–^23^ Barbera et al introduced the Edmonton Symptom Assessment Scale (ESAS), which assesses nine common cancer-related symptoms, enabling earlier symptom identification and management in the outpatient setting. This scoring system was associated with reduced ED visit rates (relative rate [RR] = 0.92; 95% CI, 0.91–0.93) and hospitalization rates (RR = 0.86; 95% CI, 0.85–0.87).^19^ Chaftari et al used procalcitonin (PCT) and lactate levels to identify febrile neutropenia patients at high risk of bloodstream infection. Procalcitonin demonstrated superior predictive performance (area under the receiver operator characteristic curve [AUC] = 0.76) compared to lactate (AUC =. 0.56; *P* = < .001) or the Multinational Association for Supportive Care in Cancer (MASCC) score (AUC = 0.65; *P* = .03).^20^ Coyne et al compared the MASCC score with the Clinical Index of Stable Febrile Neutropenia (CISNE) score for risk stratification of patients with febrile neutropenia. The CISNE score demonstrated higher specificity (98.3%) than the MASCC score (54.2%) in identifying low-risk febrile neutropenia patients.^21^

Daly et al implemented an artificial intelligence (AI)-supported monitoring system that stratified patients at therapy initiation into high- or low-risk groups for acute care needs. High-risk patients received more intensive support, which reduced ED visit rates from 0.47 to 0.27 (*P* = .01).^22^ Gajra et al developed an augmented-intelligence tool using continuous machine-learning to predict avoidable use of acute care and generate nurse-implemented, patient-specific recommendations. Monthly ED visit rate per 100 unique patients dropped from 13.7 to 11.5, and quarterly unplanned admissions from 19.7 to 17.1, although no statistical testing was reported.^23^

### Dedicated Cancer Urgent Care Facilities

Five studies evaluated dedicated cancer urgent care facilities.^24^–^28^ Ahn et al evaluated the implementation of an in-house cancer ED separate from the main ED. This intervention did not significantly affect ED LOS (31.6 to 33.7 hours [*P* = .15]).^24^ Galloway et al reported on the introduction of an urgent cancer care clinic located separately from the ED and staffed by primary care physicians. It did not significantly affect ED visits or hospitalizations.^25^ Gould Rothberg et al reported that the establishment of an oncology extended care clinic within the hospital reduced ED visits by 4.6 per 100 patients per four months (*P* = .04).^26^ Hong et al examined the implementation of an urgent care clinic for oncology patients. Weekday ED visit rate decreased (0.43 to 0.19 per 1,000 patient-months (*P* = < .001)), whereas weekend visits were unchanged (0.08 to 0.05, *P* = .53).^27^ Kuo et al assessed an off-site rapid assessment clinic functioning as an outpatient unit. It reduced median time to medical review from 40 to 28.5 minutes (*P* = .12) and significantly shortened total review time from 9.7 to 3.1 hours (*P* = < .001).^28^

### Protocolized Care

Three studies assessed protocolized care for patients with cancer, focusing on earlier antibiotic delivery in febrile neutropenia patients undergoing active cancer treatment visiting the ED.^29^–^31^ All studies measured time from ED arrival to antibiotic administration, reporting significant reductions (235 to 81 minutes (*P* = < .001),^29^ 300 to 47 minutes (*P* = < .05),^30^ and 198 to 98 minutes (*P* = < .001).^31^ Emergency department LOS decreased in two studies (6.0 to 4.4 hours (*P* = < .001),^29^ 105 to 76 minutes (*P* = .46)^30^ but increased in one (402.6 to 460.8 minutes (*P* = .13).^31^

### Staffing Optimization

Three studies evaluated the use of staff-focused interventions. Brooks et al reported on the effect of adding an evening-shift medical oncologist (5 pm – 11 pm, Sunday–Friday) The proportions of oncology patients admitted within two days of ED presentation and non-admitted patients requiring acute care within five days did not differ between the pre- and post-intervention periods.^32^ Kurtz et al evaluated the implementation of a symptom-control program involving 10 nurse contacts (five in person, five by telephone) over a 20-week chemotherapy period, compared to five contacts in usual care. Intervention patients had fewer ED visits at all time points: 0.37 vs 0.21 (baseline); 0.53 vs 0.33 (10 weeks), and 0.57 vs 0.18 (20 weeks) (ED visit coefficient: 0.254, *P* = .05).^33^ Largamente et al evaluated the introduction of an ED pathway that included a medical oncology resident and direct admission to the medical oncology department. This significantly reduced inpatient ED LOS (58 to 42 hours [*P* = .03]), inpatient LOS (15.5 to 6.5 days [*P* = < .001]), and admission rate (70 to 41% [*P* = <.001]), although ED LOS remained nearly two days.^34^

## DISCUSSION

This systematic review provides an overview of interventions aimed at optimizing ED input, throughput and output for patients with cancer. We included 16 articles (2006–2020) categorized into four research areas: scoring systems; dedicated urgent cancer care facilities; protocolized care; and staffing optimization.

### Scoring Systems

Scoring systems are mathematical models designed to support clinical decision-making at various points in the patient care pathway. The goal in using a scoring system is to reduce ED input by identifying low-risk patients suitable for outpatient management or to prevent hospitalization following an ED visit. Although most scoring systems demonstrated good predictive performance, few have been integrated into routine clinical practice. Three scoring systems used patient-reported outcomes to monitor symptoms remotely. The ESAS reduces ED visits by earlier symptom identification and outpatient management of emerging issues.^35^ A follow-up study confirmed that ESAS also predicts overall survival in patients with cancer.^36^ The ESAS is currently used to assess and manage symptom burden in palliative care settings.^37^

Several studies evaluated remote patient monitoring programs combining symptom tracking and risk scores to identify high-risk patients early and deliver timely follow-up to reduce acute care use.^22^,^23^,^38^–^40^ Only Daly et al^22^ and Gajra et al^23^ described the actual implementation of remote patient monitoring models in clinical practice and reported outcomes, showing reductions in ED visits and hospitalization rates. No subsequent studies have re-examined the effect of remote patient monitoring on ED use, although a separate study by Daly et al did show that patient engagement was high and that a daily electronic remote patient monitoring program based on patient-reported outcomes was feasible.^41^

While these findings underscore the promise of symptom-based remote monitoring, effective ED triage depends on risk scores grounded in objective physiological signs. Several scoring systems that use such signs exist for febrile neutropenia. Of these, CISNE is the most promising, having been validated and applied in multiple studies.^42^–^44^ Despite CISNE’s strong performance, MASCC score remains the clinical gold standard. Nonetheless, CISNE has been included along with MASCC in certain guidelines.^45^,^46^ However, risk stratification is not limited to patient-reported data; biochemical markers provide an additional layer of prognostic insight.

Procalcitonin is a known predictor of adverse outcomes (eg, death, intensive care unit admission) and shows a strong negative predictive value for bloodstream infection. It performs comparably with the MASCC score. Incorporating procalcitonin to established tools (MASCC, CISNE, or Early Warning Scores) improves detection of high-risk febrile neutropenia patients. However, procalcitonin has not yet been developed into a stand-alone scoring system, and most febrile neutropenia guidelines continue to rely exclusively on clinical criteria.^45^–^48^

Overall, scoring systems show strong potential to reduce ED visits and enable safe outpatient management. They offer validated, easy-to-apply metrics and are widely available, facilitating integration into clinical workflows. Across studies, these tools consistently outperformed alternative approaches, even when evaluated using diverse ED metrics.^49^,^50^ Ongoing advances in AI, including large-language models and natural language processing, are expected to further enhance scoring systems.^51^–^54^

### Dedicated Cancer Urgent Care Facilities

We defined a dedicated cancer urgent care facility as a specialized unit integrated within emergency care, designed to serve cancer patients with acute care needs. They reduce ED input by diverting patients and improve throughput by offering post-triage care in a focused setting. Implementation is rare—only one additional study has reported their use.^55^ Evidence remains inconclusive, with no long-term data. Although hospitals can tailor these units to local needs, they require significant investment in staff, space, and equipment.

### Protocolized Care

Protocolized care aims to shorten ED LOS and improve ED throughput by delivering structured, timely care. While all three studies reported positive outcomes, they focused exclusively on febrile neutropenia, even though patients with cancer often present with a broader range of acute symptoms.^56^,^57^ Symptom-specific protocols (eg, for pain crises or acute dyspnea) may offer similar benefits but remain untested. Moreover, no study published follow-up data, and only one study described actual protocol implementation.^58^

### Staffing Optimization

Staffing optimization is focused on optimizing ED throughput by specialized staffing strategies. The “nocturnal oncologist,” a senior physician working night shifts in the ED, had no significant effect, leaving its effectiveness uncertain.^32^ Two reviews noted similar strategies on reducing unplanned acute care through specialized staffing, both suggesting that such approaches often fail to achieve their intended goals.^59^,^60^ An exception appears to be the study by Legramante et al that demonstrated significant reductions in all outcomes after introducing an ED pathway with a resident and direct admission.^34^ However, ED LOS remained notably long at 42 hours, highlighting ongoing systemic challenges. Kurtz et al described the implementation of a symptom-control program that increased nurse-patient contact.^33^ Although ED visits declined, ED LOS was not measured, leaving the intervention’s direct effect on ED processes unclear. Similar nurse-led models have lowered acute-care use elsewhere, but convincing evidence for sustained improvements in ED efficiency is still lacking.^61^,^62^

### Recommendations

Future research should build on three themes. First, the rapid expansion of remote patient monitoring reflects a broader shift from clinic- to home-based surveillance. Many of the studies we reviewed described some form of remote patient monitoring, and this trend may accelerate further with the integration of vital sign monitoring into existing frameworks. Second, several promising tools remain at the prototype stage and await implementation (eg, the predictive model by Csik et al).^40^ These partially developed interventions warrant prospective validation. In addition, the socioeconomic outcomes reported in a subset of studies also merit further investigation and validation. Third, research must be paired with implementation. New solutions alone will not improve ED care for patients with cancer unless hospitals adopt them. The intervention categories mapped in this review can guide sites in selecting and rolling out the most suitable options. In our view, scoring systems are best suited to reduce ED input, while protocolized care addresses ED throughput. Implementing both—scoring systems for triage and protocolized care management—could provide the greatest benefit to patients with cancer.

## LIMITATIONS

This review summarizes interventions aimed at optimizing the input, throughput, and output in ED care for patients with cancer. To our knowledge, it is the first to focus specifically on intervention strategies tailored to this population. Previous reviews in emergency care have primarily mapped challenges, general strategies, patient characteristics, or ED-related outcomes.^63^–^65^ Oncology-focused reviews, on the other hand, mainly described presenting complaints or outcome patterns rather than concrete interventions.^66^,^67^

Several limitations should be noted. First, a meta-analysis was impossible because the included studies differed widely in design, intervention category, outcome measures, and sample size. Second, although we identified four broad intervention categories, others may have been missed. Third, limiting the review to a single-study design could have yielded a more homogeneous dataset, and would have allowed for stronger comparison between studies, but doing so would have conflicted with our exploratory objective. Finally, as the QuADS tool lacks a cut-off for the quality of a research paper, we could not assess the overall quality of included studies.

## CONCLUSION

We identified four categories of interventions to improve acute care in the emergency department for patients with cancer: scoring systems; dedicated cancer urgent care facilities; protocolized care; and staffing optimization. Rather than developing new tools, future efforts should prioritize the implementation, validation, and refinement of these existing strategies, of which scoring systems show the most potential.

## Supplementary Information



## Figures and Tables

**Table 1:** Overview of Included studies, divided per category, on interventions designed to optimize emergency department input, throughput, and output for patients with cancer.

Author (Year)	Country	Patient group (Sample size)	Study type	Intervention	Aim	Outcome(s)	Results	Level of evidence
**Scoring systems**								
Barbera (2020)	Canada	All adult patients with cancer (257,789)	Retrospective matched cohort study	Edmonton Symptom Assessment Scale (ESAS)	Evaluate the effect of ESAS on ED visits and hospitalizations	RR of ED visits	Exposed vs unexposed: ED visits 0.92	III
						RR of hospitalizations	Exposed vs unexposed: hospitalizations 0.86	
Chaftari (2021)	United States	Patients with FN attending the ED (550)	Retrospective review	PCT level with or without lactate to predict bloodstream infection	Predict high-risk bloodstream infection	Diagnostic performance of PCT levels, lactate levels, MASCC scores for the prediction of various outcomes	PCT level was a significantly better predictor of BSI than MASCC score (P = <.001) or lactate level (P = <.001)	III
Coyne (2016)	United States	Patients with FN attending the ED (230)	Retrospective cohort study	MASCC and CISNE risk-stratification scores	Compare predictive accuracy of MASCC and CISNE	Specificity in identifying low-risk FN patients	CISNE score vs MASCC score: 98.3% vs 54.2% specific	III
Daly (2022)	United States	Patients with high risk of need for acute care (81)	Cohort quality improvement study	A RPM intervention, designed to identify and monitor patients at high risk for an acute care visit.	Evaluate a digital platform that identifies and monitors high-risk patients with the goal of preventing acute care use	Cumulative incidence of ED visits	Intervention group vs control group: 0.27 vs 0.47 (P = .01)	II
						Cumulative incidence of hospitalizations	Intervention group vs control group: 0.23 vs 0.41 (P = .02)	
Gajra (2023)	United States	Patients with ED visits or hospitalizations (28,578)	Cohort quality improvement study	An augmented intelligence tool, designed to predict risk of preventable harm and generate patient-specific recommendations	Reduce acute care use using an augmented intelligence tool	Monthly ED visit rate per hundred unique patients	Pre- vs post-intervention: 13.7 vs 11.5 (no P-values reported)	II
						Quarterly unplanned admissions per hundred patients	Pre- vs post-intervention: 19.5 vs 17.1 (no P-values reported)	
**Dedicated cancer urgent care facilities**								
Ahn (2012)	South Korea	Cancer patients attending the ED (5502)	Retrospective review	Cancer emergency care unit	Evaluate the benefits of the cancer emergency care unit	ED LOS	Pre- vs post-intervention: 31.6 hours vs 33.7 hours (P = .15)	II
Galloway (2023)	Canada	Patients with cancer and serious blood disorders (18800)	Interrupted time series analysis	Urgent cancer care clinic	Examine the impact of the urgent cancer care clinic	RR of hospitalizations	Pre- vs post-intervention: 1.07 (P = .07)	III
						RR of ED visits	Pre- vs post-intervention: 0.96 (P = .51)	
						RR of ED visits during clinic hours	Pre- vs post-intervention: 1.03 (P = .58)	
Gould Rothberg (2021)	United States	Patients on active therapy (2188)	Quasi-experimental study	Oncology extended care clinic	Decrease acute care use using an oncology extended care clinic	ED visits per hundred patients	Pre- vs post-intervention: decrease of 4.6 ED visits per hundred patients (P = .04)	II
						Hospitalizations per hundred patients	Pre- vs post-intervention: Decrease of 3.29 hospitalizations per hundred patients (P = .04)	
Hong (2019)	United States	Cancer patients (33316)	Interrupted time series analysis	An urgent care clinic specifically for patients with cancer	Reduce ED visit rates using an urgent care clinic	Increase in weekday ED visit rate per month	Pre- vs post-intervention: 0.43 vs 0.19 (P = < .001)	III
						Increase in weekend ED visit rate per month	Pre- vs post-intervention: 0.08 vs 0.05 (P = .53)	
Kuo (2016)	Australia	Patients receiving chemotherapy attending the Rapid Assessment Clinic (217)	Retrospective review	Rapid Assessment Clinic	Evaluate the improvement from a Rapid Assessment Clinic	Waiting time to medical review	ED vs Rapid Assessment Clinic: 40 vs 28.5 min (P = .12)	III
						Total time spent for review	ED vs Rapid Assessment Clinic: AC: 9.7 vs 3.1 hrs. (P = < .001)	
**Protocolized care**								
Keng (2015)	Singapore	Patients attending the ED with fever (386)	Prospective cohort study	A FN pathway	Reduce antibiotic delays using a FN pathway	Time-to-antibiotics	Pre- vs post-intervention: 235 min vs 81 min (P = < .001)	II
Ko (2014)	Canada	FN patients attending the ED receiving chemotherapy within one month of ED visit (69)	Case series with internal comparison	A treatment protocol for FN patients	Evaluate the outcomes of the implemented treatment protocol	Mean door-to-antibiotics time	Intervention vs control group: 47 min vs 300 min (P = < .05)	IV
						Mean ED LOS	Intervention vs control group: 76 min vs 105 min (P = .46)	
Seltzer (2022)	United States	Adult ED FN patients (121)	Observational cohort study	An ED intervention protocol	To reduce time to initial antibiotic treatment	Time to initial antibiotic treatment	Pre- vs post-intervention: 197.6 vs 97.7 min (P = <.001)	II
**Staffing optimization**								
Brooks (2016)	United States	Patients under active outpatient management (158)	Pilot intervention	An evening-shift medical oncologist (5 PM – 11 PM, Sunday – Friday)	To reduce hospitalizations among patient with solid tumors	Proportion of eligible oncology patients admitted within two calendar days of ED presentation	Pre-intervention vs intervention period: 70% vs 69% (P = .62)	II
						Proportion of non-admitted patients who received additional acute care within 5 days of the index ED presentation.	Pre-intervention vs intervention period: 77% vs 67 % (P = .08)	
Kurtz (2006)	United States	Patients currently undergoing chemotherapy (220)	Randomized controlled trial	A symptom control intervention: a 10-contact (5 in person, 5 by telephone) 20-week nursing intervention	To reduce ED, hospital, and physician services	ED visits	Intervention group reported fewer ED visits than the control group (P = .05)	II
Legramante (2018)	United States	Patients attending the ED (250)	Prospective cohort study	A dedicated ED cancer pathway	Analyze the impact of the ED cancer pathway	Inpatient admission	Pre- vs post-intervention: 70% vs 41 % (P = <.001)	II
						ED LOS	Pre- vs post-intervention: 58 vs 42 hours (P = .03)	
						Inpatient LOS	Pre- vs post-intervention: 15.5 vs 6.5 days (P = <.001)	

*BSI*, bloodstream infection; *CISNE*, Clinical Index of Stable Febrile Neutropenia; *ED*, emergency department; *FN*, febrile neutropenia; *LOS*, length of stay; *MASCC*, Multinational Association of Supportive Care in Cancer; *PCT*, procalcitonin; *RR*, relative rate.

**Table 2:** Using the Quality Assessment with Diverse Studies criteria to evaluate studies with different designs across 13 criteria, each scored from zero (not mentioned) to three (extensively mentioned in the paper).

	Theoretical or conceptual underpinning to the research	Statement of research aim/s	Clear description of research setting and target population	The study design is appropriate to address the stated research aim/s	Appropriate sampling to address the research aim/s	Rationale for choice of data collection tool/s	The format and content of data collection tool is appropriate to address the stated research aim/s	Description of data collection procedure	Recruitment data provided	Justification for analytic method selected	The method of analysis was appropriate to answer the research aim/s	Evidence that the research stakeholders have been considered in research design or conduct	Strengths and limitations critically discussed	Total
Ahn	1	3	3	3	2	1	2	2	2	0	2	0	1	22
Barbera	2	2	3	3	3	1	3	3	2	2	3	0	1	28
Brooks	1	1	2	1	1	0	2	2	3	3	3	0	2	21
Chaftari	2	3	2	2	1	1	2	1	1	2	2	0	2	21
Coyne	3	3	2	2	1	2	3	3	3	1	3	1	2	29
Daly	1	2	3	3	1	1	2	2	3	0	2	2	1	23
Gajra	3	3	2	3	1	2	3	3	1	1	2	0	0	24
Galloway	3	3	3	2	1	2	2	2	1	3	2	1	2	27
Gould Rothberg	2	2	2	2	2	1	2	1	2	1	3	0	1	21
Hong	2	2	2	2	1	1	2	3	1	1	2	1	2	22
Keng	2	3	3	3	2	1	2	1	1	3	3	1	2	27
Ko	2	3	2	2	1	1	2	1	1	0	2	1	1	20
Kuo	3	3	2	2	2	1	2	2	1	0	1	2	1	22
Kurtz	2	2	0	1	2	1	2	3	3	3	2	2	0	23
Legramante	2	3	3	2	2	0	1	0	1	0	2	1	1	18
Seltzer	3	3	3	3	2	1	3	3	1	3	3	1	3	32

Each point was assigned a color for clarity: 0 = red, 1 = orange, 2 = yellow, 3 = green.

## References

[1] Lash RS, Bell JF, Reed SC (2017). A systematic review of emergency department use among cancer patients. Cancer Nurs.

[2] Services EA (2024). Levels of evidence in research.

[3] Alishahi Tabriz A, Turner K, Hong YR (2023). Trends and characteristics of potentially preventable emergency department visits among patients with cancer in the US. JAMA Netw Open.

[4] Morley C, Unwin M, Peterson GM (2018). Emergency department crowding: a systematic review of causes, consequences and solutions. PLoS One.

[5] Asplin BR, Magid DJ, Rhodes KV (2003). A conceptual model of emergency department crowding. Ann Emerg Med.

[6] Gallaway MS, Idaikkadar N, Tai E (2021). Emergency department visits among people with cancer: frequency, symptoms, and characteristics. J Am Coll Emerg Physicians Open.

[7] Sartini M, Carbone A, Demartini A (2022). Overcrowding in emergency department: causes, consequences, and solutions-a narrative review. Healthcare (Basel).

[8] Moher D, Liberati A, Tetzlaff J (2009). Preferred Reporting Items for Systematic Reviews and Meta-analyses: the PRISMA statement. J Clin Epidemiol.

[9] Rethlefsen ML, Kirtley S, Waffenschmidt S (2021). PRISMA-S: an extension to the PRISMA statement for reporting literature searches in systematic reviews. Syst Rev.

[10] Bramer WM, de Jonge GB, Rethlefsen ML (2018). A systematic approach to searching: an efficient and complete method to develop literature searches. J Med Libr Assoc.

[11] Harzing AW (2023). Publish or Perish.

[12] Bramer WM, Rethlefsen ML, Kleijnen J (2017). Optimal database combinations for literature searches in systematic reviews: a prospective exploratory study. Syst Rev.

[13] Bramer W (2018). Reference checking for systematic reviews using Endnote. J Med Libr Assoc.

[14] Bramer W, Bain P (2017). Updating search strategies for systematic reviews using EndNote. J Med Libr Assoc.

[15] Bramer WM, Giustini D, de Jonge GB (2016). De-duplication of database search results for systematic reviews in EndNote. J Med Libr Assoc.

[16] Ouzzani M, Hammady H, Fedorowicz Z (2016). Rayyan—a web and mobile app for systematic reviews. Systematic Reviews.

[17] Services EA Levels of evidence in research.

[18] Harrison R, Jones B, Gardner P (2021). Quality Assessment with Diverse Studies (QuADS): an appraisal tool for methodological and reporting quality in systematic reviews of mixed- or multi-method studies. BMC Health Services Research.

[19] Barbera L, Sutradhar R, Seow H (2020). Impact of standardized Edmonton Symptom Assessment System use on emergency department visits and hospitalization: results of a population-based retrospective matched cohort analysis. JCO Oncol Pract.

[20] Chaftari P, Chaftari AM, Hachem R (2021). The role of procalcitonin in identifying high-risk cancer patients with febrile neutropenia: a useful alternative to the multinational association for supportive care in cancer score. Cancer Med.

[21] Coyne CJ, Le V, Brennan JJ (2017). Application of the MASCC and CISNE risk-stratification scores to identify low-risk febrile neutropenic patients in the emergency department. Ann Emerg Med.

[22] Daly B, Nicholas KJ, Flynn J (2022). Association between remote monitoring and acute care visits in high-risk patients initiating intravenous antineoplastic therapy. JCO Oncol Pract.

[23] Gajra A, Jeune-Smith Y, Balanean A (2023). Reducing avoidable emergency visits and hospitalizations with patient risk-based prescriptive analytics: a quality improvement project at an oncology care model practice. JCO Oncol Pract.

[24] Ahn S, Lee YS, Lim KS (2012). Emergency department cancer unit and management of oncologic emergencies: experience in Asan medical center. Support Care Cancer.

[25] Galloway K, Lambert P, Bow EJ (2023). Evaluation of the impact of the urgent cancer care clinic on emergency department visits, primary care clinician visits, and hospitalizations in Winnipeg, Manitoba. Curr Oncol.

[26] Gould Rothberg BE, Canavan ME, Mun S (2022). Impact of a dedicated cancer urgent care center on acute care utilization. JCO Oncol Pract.

[27] Hong AS, Froehlich T, Clayton Hobbs S (2019). Impact of a cancer urgent care clinic on regional emergency department visits. J Oncol Pract.

[28] Kuo JC, De Silva M, Diwakarla C (2017). A rapid access clinic to improve delivery of ambulatory care to cancer patients. Asia Pac J Clin Oncol.

[29] Keng MK, Thallner EA, Elson P (2015). Reducing time to antibiotic administration for febrile neutropenia in the emergency department. J Oncol Pract.

[30] Ko HF, Tsui SS, Tse JW (2015). Improving the emergency department management of post-chemotherapy sepsis in haematological malignancy patients. Hong Kong Med J.

[31] Seltzer JA, Frankfurt O, Kyriacou DN (2022). Association of an emergency department febrile neutropenia intervention protocol with time to initial antibiotic treatment. Acad Emerg Med.

[32] Brooks GA, Chen EJ, Murakami MA (2016). An ED pilot intervention to facilitate outpatient acute care for cancer patients. Am J Emerg Med.

[33] Kurtz ME, Kurtz JC, Given CW (2006). Effects of a symptom control intervention on utilization of health care services among cancer patients. Med Sci Monit.

[34] Legramante JM, Pellicori S, Magrini A (2018). Cancer patients in the emergency department: a “nightmare” that might become a virtuous clinical pathway. Anticancer Res.

[35] Caissie A, Olson R, Barbera L (2022). Striving to fill in gaps between clinical practice and standards: the evolution of a Pan-Canadian approach to patient-reported outcomes use. Curr Oncol.

[36] Barbera L, Sutradhar R, Seow H (2020). The impact of routine Edmonton Symptom Assessment System (ESAS) use on overall survival in cancer patients: results of a population-based retrospective matched cohort analysis. Cancer Med.

[37] Alsuliman HR, Alsaigh SA, Habib FA (2024). Exploring the influence of the Edmonton Symptom Assessment System implementation in palliative care patients: a systematic review. Cureus.

[38] Grant RC, Moineddin R, Yao Z (2019). Development and validation of a score to predict acute care use after initiation of systemic therapy for cancer. JAMA Netw Open.

[39] Peterson DJ, Ostberg NP, Blayney DW (2021). Machine learning applied to electronic health records: identification of chemotherapy patients at high risk for preventable emergency department visits and hospital admissions. JCO Clin Cancer Inform.

[40] Csik VP, Li M, Binder AF (2021). Development of an oncology acute care risk prediction model. JCO Clinical Cancer Informatics.

[41] Daly B, Nicholas K, Flynn J (2022). Analysis of a remote monitoring program for symptoms among adults with cancer receiving antineoplastic therapy. JAMA Netw Open.

[42] Monuszko KA, Albright B, Montes De Oca MK (2021). Evaluation of the Clinical Index of Stable Febrile Neutropenia risk stratification system for management of febrile neutropenia in gynecologic oncology patients. Gynecol Oncol Rep.

[43] Moon H, Choi YJ, Sim SH (2018). Validation of the Clinical Index of Stable Febrile Neutropenia (CISNE) model in febrile neutropenia patients visiting the emergency department. Can it guide emergency physicians to a reasonable decision on outpatient vs. inpatient treatment?. PLoS One.

[44] Ono Y, Hayama N, Hattori S (2022). Can MASCC and CISNE scores predict delays of lung cancer chemotherapy after febrile neutropenia?. Thorac Cancer.

[45] Taplitz RA, Kennedy EB, Bow EJ (2018). Outpatient management of fever and neutropenia in adults treated for malignancy: American Society of Clinical Oncology and Infectious Diseases Society of America Clinical Practice Guideline update. J Clin Oncol.

[46] Coyne CJ, Castillo EM, Shatsky RA (2022). Procalcitonin as a predictive tool for death and ICU admission among febrile neutropenic patients visiting the emergency department. Medicina (Kaunas).

[47] Garcia de Guadiana-Romualdo L, Cerezuela-Fuentes P, Espanol-Morales I (2019). Prognostic value of procalcitonin and lipopolysaccharide binding protein in cancer patients with chemotherapy-associated febrile neutropenia presenting to an emergency department. Biochem Med (Zagreb).

[48] Coyne CJ, Castillo EM, Shatsky RA (2022). Procalcitonin as a predictive tool for death and ICU admission among febrile neutropenic patients visiting the emergency department. Medicina (Kaunas).

[49] Lu SC, Knafl M, Turin A (2023). Machine learning models using routinely collected clinical data offer robust and interpretable predictions of 90-day unplanned acute care use for cancer immunotherapy patients. JCO Clin Cancer Inform.

[50] Grant RC, He JC, Khan F (2023). Machine learning-based early warning systems for acute care utilization during systemic therapy for cancer. J Natl Compr Canc Netw.

[51] Liefers B (2025). The impact of artificial intelligence on predictive modeling in clinical trials. J Evol Med.

[52] Meijerink LM, Dunias ZS, Leeuwenberg AM (2025). Updating methods for artificial intelligence-based clinical prediction models: a scoping review. J Clin Epidemiol.

[53] Brann F, Sterling NW, Frisch SO (2024). Sepsis prediction at emergency department triage using natural language processing: retrospective cohort study. JMIR AI.

[54] Lyman GH, Kuderer NM (2024). Artificial intelligence and cancer clinical research: III risk prediction models for febrile neutropenia in patients receiving cancer chemotherapy. Cancer Invest.

[55] Carlson LC, Raja AS, Dworkis DA (2020). Impact of urgent care openings on emergency department visits to two academic medical centers within an integrated health care system. Ann Emerg Med.

[56] Koenig C, Schneider C, Morgan JE (2020). Interventions aiming to reduce time to antibiotics (TTA) in patients with fever and neutropenia during chemotherapy for cancer (FN), a systematic review. Support Care Cancer.

[57] Casanovas Blanco M (2019). Critical review of emergency department management of chemotherapy complications in cancer patients. Eur J Cancer Care (Engl).

[58] Melikian L, Bullington S, Harris B (2023). Implementation of a protocol for management of febrile neutropenia in the emergency department at Veteran Health Indiana. Fed Pract.

[59] Handley NR, Schuchter LM, Bekelman JE (2018). Best practices for reducing unplanned acute care for patients with cancer. J Oncol Pract.

[60] Cooksley T, Rice T (2017). Emergency oncology: development, current position and future direction in the USA and UK. Support Care Cancer.

[61] Bischof JJ, Bush M, Shams RB (2021). A hybrid model of acute unscheduled cancer care provided by a hospital-based acute care clinic and the emergency department: a descriptive study. Support Care Cancer.

[62] Krzyzanowska MK, MacKay C, Han H (2019). Ambulatory Toxicity Management (AToM) pilot: results of a pilot study of a pro-active, telephone-based intervention to improve toxicity management during chemotherapy for breast cancer. Pilot Feasibility Stud.

[63] Austin EE, Blakely B, Tufanaru C (2020). Strategies to measure and improve emergency department performance: a scoping review. Scand J Trauma Resusc Emerg Med.

[64] Burgess L, Ray-Barruel G, Kynoch K (2022). Association between emergency department length of stay and patient outcomes: a systematic review. Res Nurs Health.

[65] Berg E, Weightman AT, Druga DA (2020). Emergency department operations II: patient flow. Emerg Med Clin North Am.

[66] Nene RV, Brennan JJ, Castillo EM (2021). Cancer-related emergency department visits: comparing characteristics and outcomes. West J Emerg Med.

[67] Gould Rothberg BE, Quest TE, Yeung SJ (2022). Oncologic emergencies and urgencies: a comprehensive review. CA Cancer J Clin.

