# Supplementary material for: Systematic Review of Interventions to Optimize Emergency Department Care of Patients with Cancer
Source: West J Emerg Med. 2026 Feb 22;27(2):269–80. doi: 10.5811/westjem.49006 (PMC13016046; doi:10.5811/westjem.49006)
Supplement: Supplementary file 1 [file wjem-27-269-s001.docx]

**APPENDIX 1: Full Query**

| **Database searched** | **Platform** | **Years of coverage** | **Records** | **Records after duplicates removed** |
| --- | --- | --- | --- | --- |
| Medline ALL | Ovid | 1946 - Present | 2671 | 2665 |
| Embase | Embase.com | 1971 - Present | 5714 | 3667 |
| Web of Science Core Collection* | Web of Knowledge | 1975 - Present | 2296 | 507 |
| Cochrane Central Register of Controlled Trials | Wiley | 1992 - Present | 476 | 334 |
| Additional Search Engines: Google Scholar** (200 top-ranked) | | | 200 | 142 |
| **Total** | | | **11357** | **7315** |

*Science Citation Index Expanded (1975-present) ; Social Sciences Citation Index (1975-present) ; Arts & Humanities Citation Index (1975-present) ; Conference Proceedings Citation Index- Science (1990-present) ; Conference Proceedings Citation Index- Social Science & Humanities (1990-present) ; Emerging Sources Citation Index (2005-present)

**Google Scholar was searched via "Publish or Perish" to download the results in EndNote.

No other database limits were used than those specified in the search strategies exclude conference abstracts only English

**Embase**

('emergency ward'/de OR 'oncologic emergency'/de OR emergency/de OR 'out-of-hours care'/de OR 'emergency care'/de OR 'emergency health service'/de OR 'hospital emergency service'/de OR (((emergency OR A&E OR A-E OR "A and E" OR "accident and emergency" OR urgent-care* OR out-of-hours* OR after-hours*) NEAR/3 (ward* OR unit* OR department* OR room* OR admission* OR cent* OR clinic OR clinics)) OR (accident NEXT/2 emergency) OR ((acute OR emergency) NEXT/2 (care*)) OR oncol*-emergenc*):ab,ti,kw) **AND** ('malignant neoplasm'/exp OR 'cancer patient'/de OR (cancer* OR neoplas* OR tumor* OR tumour* OR malignan*):ab,ti,kw) **AND** ('intervention study'/de OR 'medical service'/de OR 'clinical pathway'/de OR 'organizational efficiency'/exp OR 'length of stay'/de OR 'health care delivery'/de OR 'health care utilization'/exp OR 'waiting time'/de OR (((intervent*) NEAR/3 (stud* OR trial*)) OR ((medical OR oncolog* OR acute) NEAR/3 (service*)) OR ((additional OR addition-of) NEAR/3 (doctor* OR unit* OR service* OR intervent* OR room* OR program*)) OR ((clinical OR patient*) NEAR/3 (pathway* OR path OR paths)) OR ((patient* OR oncolog*) NEAR/3 (service* OR program*)) OR length-of-stay* OR LOS OR ((deliver* OR utili* OR efficien*) NEAR/3 (care* OR healthcare* OR health* OR organization* OR organisation*)) OR ((wait*) NEAR/3 (time*))):ab,ti,kw OR intervent*:ti) NOT ([Conference Abstract]/lim OR [Conference Review]/lim) AND [ENGLISH]/lim

**Medline**

(exp Emergency Service, Hospital/ OR Emergencies/ OR After-Hours Care/ OR Emergency Medical Services/ OR exp Emergency Service, Hospital/ OR (((emergency OR A&E OR A-E OR "A and E" OR "accident and emergency" OR urgent-care* OR out-of-hours* OR after-hours*) ADJ3 (ward* OR unit* OR department* OR room* OR admission* OR cent* OR clinic OR clinics)) OR (accident ADJ2 emergency) OR ((acute OR emergency) ADJ2 (care*)) OR oncol*-emergenc*).ab,ti,kf.) **AND** (exp Neoplasms/ OR (cancer* OR neoplas* OR tumor* OR tumour* OR malignan*).ab,ti,kf.) **AND** (exp Clinical Trial/ OR Critical Pathways/ OR Efficiency, Organizational/ OR Length of Stay/ OR Delivery of Health Care/ OR Patient Acceptance of Health Care/ OR Waiting Lists/ OR (((intervent*) ADJ3 (stud* OR trial*)) OR ((medical OR oncolog* OR acute) ADJ3 (service*)) OR ((additional OR addition-of) ADJ3 (doctor* OR unit* OR service* OR intervent* OR room* OR program*)) OR ((clinical OR patient*) ADJ3 (pathway* OR path OR paths)) OR ((patient* OR oncolog*) ADJ3 (service* OR program*)) OR length-of-stay* OR LOS OR ((deliver* OR utili* OR efficien*) ADJ3 (care* OR healthcare* OR health* OR organization* OR organisation*)) OR ((wait*) ADJ3 (time*))).ab,ti,kf. OR intervent*.ti.) NOT (news OR congres* OR abstract* OR book* OR chapter* OR dissertation abstract*).pt. AND english.la.

**Cochrane**

((((emergency OR A&E OR A NEXT E OR "A and E" OR "accident and emergency" OR urgent NEXT care* OR out NEXT of NEXT hours* OR after NEXT hours*) NEAR/3 (ward* OR unit* OR department* OR room* OR admission* OR cent* OR clinic OR clinics)) OR (accident NEXT/2 emergency) OR ((acute OR emergency) NEXT/2 (care*)) OR oncol* NEXT emergenc*):ab,ti) **AND** ((cancer* OR neoplas* OR tumor* OR tumour* OR malignan*):ab,ti) **AND** ((((intervent*) NEAR/3 (stud* OR trial*)) OR ((medical OR oncolog* OR acute) NEAR/3 (service*)) OR ((additional OR addition NEXT of) NEAR/3 (doctor* OR unit* OR service* OR intervent* OR room* OR program*)) OR ((clinical OR patient*) NEAR/3 (pathway* OR path OR paths)) OR ((patient* OR oncolog*) NEAR/3 (service* OR program*)) OR length NEXT of NEXT stay* OR LOS OR ((deliver* OR utili* OR efficien*) NEAR/3 (care* OR healthcare* OR health* OR organization* OR organisation*)) OR ((wait*) NEAR/3 (time*))):ab,ti OR intervent*:ti) NOT "conference abstract":kw

**Web of Science**

(TS=((((emergency OR A&E OR A-E OR "A and E" OR "accident and emergency" OR urgent-care* OR out-of-hours* OR after-hours*) NEAR/2 (ward* OR unit* OR department* OR room* OR admission* OR cent* OR clinic OR clinics)) OR (accident NEAR/2 emergency) OR ((acute OR emergency) NEAR/2 (care*)) OR oncol*-emergenc*) **AND** (cancer* OR neoplas* OR tumor* OR tumour* OR malignan*))) **AND** (TS=(((intervent*) NEAR/2 (stud* OR trial*)) OR ((medical OR oncolog* OR acute) NEAR/2 (service*)) OR ((additional OR addition-of) NEAR/2 (doctor* OR unit* OR service* OR intervent* OR room* OR program*)) OR ((clinical OR patient*) NEAR/2 (pathway* OR path OR paths)) OR ((patient* OR oncolog*) NEAR/2 (service* OR program*)) OR length-of-stay* OR LOS OR ((deliver* OR utili* OR efficien*) NEAR/2 (care* OR healthcare* OR health* OR organization* OR organisation*)) OR ((wait*) NEAR/2 (time*))) OR TI=(intervent*)) NOT DT=(Meeting Abstract OR Meeting Summary) AND LA=(English)

**Google Scholar**

'emergency ward|unit|department|room|clinic' cancer|neoplasm|tumor|tumour|malignancy 'intervention study|trial'|'medical|oncology|acute service'|'clinical|patient pathway|path'|'patient|oncology service|programme'|'length of stay'|'wait|waiting time'
